# Concerns about Direct Potable Reuse

**DOI:** 10.1289/ehp.1509914

**Published:** 2015-06-01

**Authors:** Brenda Adelman

**Affiliations:** Russian River Watershed Protection Committee

According to Dahl (2014), water shortages in parts of the United States are so dire that attitudes toward wastewater reuse, including direct injection into drinking water, are becoming more favorable. He noted 12 locations nationwide that directly or indirectly blend highly treated wastewater with potable supplies, with more projects planned. California’s ongoing crisis-level drought has convinced some citizens to be more open toward direct potable reuse as a viable way to expand existing supplies. As evidence of growing acceptance, Dahl quoted Daniel Nix, operations manager for Wichita Falls Public Works, stating that new regulations were developed to ensure complete protection of public health, that users reported the water tasted great, and that “[t]he quality’s good, nobody’s gotten sick, and we haven’t had any problem with the plants” (Dahl 2014).

Yet, nobody knows the extent to which potentially toxic chemicals remaining in rivers and drinking water supplies after advanced treatment may compromise health, development, and reproductive capabilities of humans, birds, fish, aquatic organisms, and other species. Many experts believe even the most rigorous water treatment technologies, including reverse osmosis and advanced membrane technology, allow low levels of contaminants to remain. Naik (2014), describing regulatory gaps and variations in irrigation requirements nationwide, stated, “Currently, there is no single treatment process that can provide a complete barrier to all chemicals.”

This is a particular concern in regards to endocrine-disrupting chemicals (EDCs). Approximately 1,000 EDCs have been identified over the last 20 years (U.S. Food and Drug Administration 2015), while more than 28 years have passed since the last chemical risk review by the U.S. Environmental Protection Agency (Jones 2014). Even minute doses of EDCs may be harmful to both humans and wildlife (Diamanti-Kandarakis et al. 2009; Zoeller et al. 2012). Unlike other toxicants, EDCs do not obey the common assumption that “the dose makes the poison” (Vandenberg et al. 2012), a fact that is not recognized by California wastewater reuse requirements (California Environmental Protection Agency 2013).

The Clean Water Act is charged to protect all beneficial uses of U.S. waters, but currently, neither this nor any other federal law regulates direct potable reuse (U.S. Environmental Protection Agency 2012). Furthermore, the lack of intervening environmental buffers in direct potable reuse—which traditionally provide mixing, dilution, and natural physical, chemical, and biological processes to protect water quality—will not allow critical time needed for corrective action in the event of an emergency (Crook 2010). Any type of disastrous event or equipment breakdown could conceivably contaminate water supplies for millions of humans and wildlife alike, especially as aging infrastructure deteriorates.
